# Survey on the Presence of Bacterial and Parasitic Zoonotic Agents in the Feces of Wild Birds

**DOI:** 10.3390/vetsci8090171

**Published:** 2021-08-25

**Authors:** Valentina Virginia Ebani, Lisa Guardone, Fabrizio Bertelloni, Stefania Perrucci, Alessandro Poli, Francesca Mancianti

**Affiliations:** 1 Department of Veterinary Sciences, University of Pisa, Viale delle Piagge 2, 56124 Pisa, Italy; lisa.guardone@vet.unipi.it (L.G.); fabrizio.bertelloni@unipi.it (F.B.); stefania.perrucci@unipi.it (S.P.); alessandro.poli@unipi.it (A.P.); francesca.mancianti@unipi.it (F.M.); 2 Centre for Climate Change Impact, University of Pisa, Via del Borghetto 80, 56124 Pisa, Italy

**Keywords:** wild avifauna, zoonosis, *Encephalitozoon*, *Salmonella*, *Coxiella burnetii*

## Abstract

Wild avifauna may act as fecal source of bacterial and parasitic pathogens for other birds and mammals. Most of these pathogens have a relevant impact on human and livestock health which may cause severe disease and economic loss. In the present study, the fecal samples collected from 121 wild birds belonging to 15 species of the genera *Anas, Tadorna, Fulica, Arddea, Larus, Falco, Athene, Accipiter*, and *Columba* were submitted to bacteriological and molecular analyses to detect *Brucella* spp., *Coxiella burnetii*, *Mycobacterium* spp., *Salmonella* spp., *Cryptosporidium* spp., *Giardia* spp., and microsporidia. Four (3.3%) animals were positive for one pathogen: one *Anas penelope* for *C. burnetii*, one *Larus michahellis* for *S. enterica* serovar Coeln, and two *Columba livia* for *Encephalitozoon hellem*. Although the prevalence rates found in the present survey were quite low, the obtained results confirm that wild birds would be the a potential fecal source of bacterial and parasitic zoonotic pathogens which sometimes can also represent a severe threat for farm animals.

## 1. Introduction

Wild avifauna includes several bird species with different features related to behaviors, habitats, feeding. All wild birds can harbor pathogens in their intestinal tract and consequently excrete these agents in their feces, thus they may be a source of infection for other birds. Furthermore, wild birds can excrete agents responsible for infectious and/or parasitic diseases in mammals, including humans. Considering that these animals often reach and live in farm areas, they may act as source of pathogens for livestock too, and cause relevant economic loss. The role of birds as vectors of disease transmission to domestic livestock has been attributed to environmental contamination of, amongst others, water supplies, pastureland, and feed by avian feces [1,2,3,4,5].

Among bacterial agents, *Brucella* spp., *Mycobacterium* spp., *Coxiella burnetii*, and *Salmonella enterica* are the most relevant zoonotic pathogens able to cause serious diseases in livestock, mainly ruminants, even though other bacterial agents (e.g., *Campylobacter* spp., *Staphylococcus* spp, *Chlamydia* spp., and *Escherichia coli*) may compromise the animal health status. Members of genus *Brucella* are Gram-negative, facultative intracellular bacteria which infect several mammal domestic and wild species; brucellosis is a relevant concern for livestock health in which the pathogen, mainly *B. abortus* and *B. melitensis*, causes abortion and infertility [6]. *Brucella* spp. have not been isolated from birds, but anti-*Brucella* antibodies have been detected in some avian species in South Africa and Asia [7,8,9,10,11,12].

Genus *Mycobacterium* includes acid-fast bacilli classified into the group of mycobacteria causing tuberculosis, such as *M. tuberculosis* and *M. bovis*, and the non-tuberculous mycobacteria (NTM) group. Among NTM, members of the *M avium* complex represent a serious threat in veterinary medicine. In particular, *M. avium avium* causes avian tuberculosis, but it is often involved in mammal infections, mainly in human, cattle, and swine [13]. Moreover, *M. genavense*, a well-known human pathogen, has been frequently found in avian population [14].

*C. burnetii* is a Gram-negative, intracellular obligate bacterium which may infect several avian and mammal species. It is the etiologic agent of the zoonotic disease Q Fever, which causes reproductive disorders mainly in farm ruminants [15].

*S. enterica*, a Gram-negative bacterium of the family Enterobacteriaceae, infects domestic and wild birds in which it causes different forms in relation to the involved serovar. *S. enterica* serovars, Gallinarum and Pullorum, cause systemic disease mainly in poultry and are not pathogen for mammals [16]. The non-specific-host serovars may infect avian populations without inducing disease, whereas they are responsible for enteric, septicemic, and reproductive diseases in several mammal species including human and farm animals [17].

Among parasites, protozoans, including *Giardia* spp. and *Cryptosporidium* spp., are known to be possibly excreted in birds’ feces [4]. *Giardia* spp. and *Cryptosporidium* spp. are usually zoonotic enteric protozoan parasites that can infect a wide range of vertebrate hosts, including humans, mammals, and domestic and wild animals worldwide. They are both widespread in wild birds too [18].

Two species of *Giardia, G. ardeae* and *G. psittaci,* have been identified in birds based on the morphology of trophozoites and cysts [19]. Beside them, other species/assemblages have also been reported from avian hosts, including the zoonotic assemblages A and B [19]. In more detail, *G. duodenalis* assemblage A was found in Brazil [20] while *G. duodenalis* assemblage B, D, and F in northwest Spain [21]. 

Presently, four *Cryptosporidium* species, distinguished on the basis of biological and genetic differences, have been reported to cause infection in birds: *C. meleagridis*, *C. baileyi*, *C. avium*, and *C. galli*. In addition, the presence of other species, including *C. andersoni*, *C. parvum*, *C. hominis*, *C. muris*, and several genotypes, such as *Cryptosporidium* goose genotypes I–IV, a *Cryptosporidium* duck genotype, and *Cryptosporidium* avian genotypes I–IV, has also been described [18,22]. In general, many of these *Cryptosporidium* species and genotypes are host-specific and, thus, are usually not considered a public health concern. However, some birds may carry and disseminate zoonotic species [23] and, in addition, *C. meleagridis* is considered the third most prevalent species known to infect humans after *C. hominis* and *C. parvum* [12,23,24].

Beside *Cryptosporidium* spp. and *Giardia* spp. several microsporidia, such as *Enterocytozoon bieneusi*, *Encephalitozoon intestinalis*, and *Encephalitozoon hellem*, are zoonotic pathogens affecting primarily immunocompromised persons [25,26,27], which have been repeatedly reported from birds [4,28,29,30,31].

Data about the potential role of wild avifauna as a fecal source of bacteria and parasites for humans and other mammals are not numerous, and in particular those concerning Italy are very scanty [32,33,34,35,36].

The aim of the present survey was to specifically verify the occurrence of some among the most important zoonotic bacterial and parasitic pathogens, which can also affect ruminant livestock, in feces collected from wild birds belonging to different orders and species. In particular, molecular analyses were carried out to detect *Mycobacterium* spp., *Brucella* spp., *Coxiella burnetii*, *Cryptosporidium* spp., *Giardia* spp., and microsporidia. Furthermore, bacteriological analyses were executed to isolate *Salmonella* spp.

## 2. Materials and Methods

### 2.1. Animals

Intestinal samples were collected from 121 free-roaming wild birds from January to December 2016. Fifty-six samples were collected from animals hunted during the hunting season in different wet areas of Central Italy. The evisceration was performed by hunters who collaborated with the authors for a previous research [34]. The remaining 65 samples were collected from birds dead at an avian recovery center located in Central Italy. No lesions ascribable to infectious and/or parasitic diseases were observed, whereas fatal traumatic lesions were considered as the cause of death. During the necropsies, a portion of terminal intestine, approximatively from caeca to cloaca, were collected from each bird and stored at 4 °C for 24–48 h until the end of the investigations.

All samples were collected from the following avian species: common teal *Anas crecca* (*n* = 22), mallard *Anas platyrhynchos* (*n* = 15), Eurasian wigeon *Anas penelope* (*n* = 11), Northern shoveler *Anas clypeata* (*n* = 3), pintail *Anas acuta* (*n* = 1), grey heron *Ardea cinerea* (*n* = 2), yellow-legged gull *Larus michahellis* (*n* = 35), common shelduck *Tadorna tadorna* (*n* = 3), Eurasian coot *Fulica atra* (*n* = 1), common kestrel *Falco tinninculus* (*n* = 3), peregrine falcon *Falco peregrinus* (*n* = 1), little owl *Athene noctua* (*n* = 1), Eurasian sparrowhawk *Accipiter nisus* (*n* = 1), common pigeon *Columba livia* (*n* = 21), common wood pigeon *Columba palumbus* (*n* = 1).

### 2.2. Ethical Statement

Regularly hunted and naturally dead birds were used in the study. No birds were sacrificed for the study.

### 2.3. Bacteriological Analyses

*Salmonella* spp. isolation was executed from each fecal sample following the procedures previously described [37]. Briefly, about 3 gr of feces was incubated in 10 mL of buffered peptone water at 37 °C for 24 h. One ml of this culture was transferred into ten mL of Selenite Cystine Broth (Oxoid Ltd., Basingstoke, UK) and one ml into ten mL of Rappaport Vassiliadis Broth. The tubes were incubated at 37 °C for 24 h and at 42 °C for 24 h, respectively. One loopful from each broth culture was streaked onto Salmonella-Shigella Agar (Oxoid) and Brilliant Green Agar (Oxoid) plates. After incubation of the plates at 37 °C for 24 h, suspected colonies were submitted to biochemical characterization and serotyping.

DNA was extracted from about 25 mg of each fecal sample using the commercial kit Tissue Genomic DNA Extraction Kit (Fisher Molecular Biology, Trevose, PA, USA) and following the procedures reported by the producer. DNA samples were kept at 4 °C, for 10 days, until used in the different PCR (Polymerase Chain Reaction) assays.

Target genes, primers sequences and PCR conditions are reported in Table 1.

All PCR amplifications were executed using the EconoTaq PLUS 2x Master Mix (Lucigen Corporation, Middleton, WI, USA) and the automated thermal cycler Gene-Amp PCR System 2700 (Perkin Elmer, Norwalk, CT, USA).

PCR products were analysed by electrophoresis on 1.5% agarose gel stained with GelRed^®^ Nucleic Acid Gel Stain (Biotium, Fremont, CA). SharpMass™ 100 Plus Ladder (Euroclone, Milano, Italy) was used as a DNA marker. 

PCR products of the expected length for microsporidia and with a sufficient concentration were forward and reverse Sanger sequenced by an external company (Eurofins Genomics, Ebersberg bei München, Germany). Nucleotide sequences were analysed using Bioedit version 7.0.9 [38]. Adjustments were made after visual checking and consensus sequences were compared against those deposited in GenBank by using the National Center for Biotechnology Information (NCBI) Basic Local Alignment Search Tool (BLAST).

**Table 1:** PCR primers and conditions employed in the assays for the detection of each pathogen. The PCR conditions refers to the cycling phase which was anticipated by 5 min at 95 °C and followed by 10 min at 72 °C. A nested PCR was used from *Cryptosporidium* spp. and a semi-nested (different forward primers and same reverse) for *Giardia* spp.

Pathogens	Amplicons (Target Gene)	Primers Sequence (5′—3′)	PCR Conditions	References
*Brucella* spp.	905 bp (16SrRNA)	F4 (TCGAGCGCCCGCAAGGGG) R2 (AACCATAGTGTCTCCACTAA)	95 °C—30 s 54 °C—90 s 72 °C—90 s For 50 cycles	[39]
*Coxiella burnetii*	687 bp (IS1111a)	Trans-1 (TATGTATCCACCGTAGCCAGT) Trans-2 (CCCAACAACACCTCCTTATTC)	95 °C—30 s 64 °C—1 min 72 °C—1 min For 40 cycles	[40]
*Mycobacterium* spp.	1030 bp (16SrDNA)	MycogenF (AGAGTTTGATCCTGGCTCAG) MycogenR (TGCACACAGGCCACAAGGGA)	95 °C—1 min 62 °C—2 min 72 °C—1 min For 40 cycles	[41]
*Cryptosporidium* spp.	1325 bp (1st step) 826-864 bp (2nd step) (16SrDNA)	outcryF (TTCTAGAGCTAATACATGCG) outcryR (CCCATTTCCTTCGAAACAGGA) incryF (GGAAGGGTTGTATTTATTAGATAAAG) incryR (AAGGAGTAAGGAACAACCTCCA)	94 °C—45 s 55 °C—45 s 72 °C—1 min For 35 cycles (1st and 2nd step)	[42]
*Giardia* spp.	432 bp (2nd step) (gdh)	GDHeF (TCAACGTYAAYCGYGGYTTCCGT) GDHiR (GTTRTCCTTGCACATCTCC) GDHiF (CAGTACAACTCYGCTCTCGG)	94 °C—1 min 56 °C—20 s 72 °C—45 s For 45 cycles	[43]
Microsporidia (*Encephalitozoon* spp. and *Enterocitozoon* spp.)	250–280 pb (18SrRNA)	V1 (CACCAGGTTGATTCTGCCTGAC) PMP2 (CCTCTCCGGAACCAAACCCTG)	94 °C—30 s 60 °C—30 s 72 °C—30 s For 35 cycles	[44]

## 3. Results

Among the analysed samples, 4 (3.3%) resulted positive for at least one pathogen (Table 2). No animals were positive for *Mycobacterium* spp., *Giardia* spp. and *Cryptosporidium* spp. One hunted *A. penelope* was positive for *C. burnetii*, one *L. michahellis,* from the recovery center, for *S. enterica* serovar Coeln and two *C. livia,* both from the recovery center, were positive for *Encephalitozoon hellem*.

## 4. Discussion

Even though the investigation was carried out on a small number of birds and very few individuals of some species, the results obtained in the present survey suggested that wild birds are not frequently important fecal spreaders of the investigated bacterial and parasitic pathogens responsible for livestock infections. 

All birds were PCR negative for *Brucella* spp. and this finding is in agreement to other previous surveys. In facts, even though some investigations found serological positive reactions in chickens, pigeons and ducks in some areas of Asia and South Africa, *Brucella* spp. was never detected so far [7,8,9,10,11,12]. Only Najadenski et al. [45] found in Bulgaria one (0.15%) *Acrocephalus arundinaceus* PCR positive for *Brucella* spp. among 706 examined wild birds migrating along the Mediterranean-Black Sea Flyway. The role of birds in the epidemiology of brucellosis is kept under control, because, even if they do not develop disease, they could act as vectors of brucellae mainly in geographic areas where this infection is largely widespread [46].

No birds were positive for *Mycobacterium* genus. All avian species are susceptible to *M. avium avium*, but the disease is rarely observed in poultry. Avian tuberculosis is most frequently observed in particular cases: birds kept in zoological gardens and cage birds that, moreover, are susceptible to *M. bovis* and *M. tuberculosis*, too [47]. Wild avian species may contract mycobacteria from the environment and they can excrete these pathogens in their feces becoming source of infection for other birds and/or mammals [14]. However, data about *Mycobacterium* infections in wild birds are limited to the description of some cases, mainly due to *M. avium avium, M. intracellulare* and *M. genavense*, but prevalence values in different geographic areas are not available.

One *A. penelope* was positive to *C. burnetii*. This pathogen can infect mammals, in which it may cause disease, as well as birds that are asymptomatic. Data about the spreading of *C. burnetii* in avian populations are very scanty [48,49,50,51,52,53]. Previous surveys carried out in Italy detected *C. bunetii* in wild avifauna with prevalence rates ranging from 3% in water fowl [54] to 5.95% in pigeons [55]. In both cases, spleen specimens were analyzed, thus the findings suggested that birds were potential source of infections, but they did not show that the tested animals were shedders of the pathogen. The present survey shows that birds, even though not frequently, may excrete *C. burnetii* in their droppings and consequently contaminate the environment.

Wild birds have been suggested to be involved in the epidemiology of bacterial enteropathogens worldwide [56,57] as well as in Italy [35]. Different *Salmonella* serovars have been isolated, thus it seems that there is no correlation between wild birds and a given serovar. In our survey *S. enterica* serovar Coeln was isolated from a gull *(L. michaellis*); this serovar resulted present in Italian wild fauna in a quite recent study that found it in wild boars [58]. However, *S.* Coeln is a rarely notified non-typhoid serovar of *Salmonella* [59,60]. Our findings confirm that gulls are involved in the epidemiology of enteropathogen bacteria [34]; in fact, they are scavenger birds largely present in different environments where they can acquire and/or excrete pathogens.

As regards parasites, no birds were positive for *Giardia* spp. nor for *Cryptosporidium* spp. This could be explained considering that low prevalence rates had already been observed for these protozoans in birds [20,22,61] and the relatively low number of samples for some avian species in the present survey. These two parasites, which are prevalent in livestock and wild animals, have also attracted attention in domestic, caged, ornamental, companion, and wild birds [18]. Cryptosporidiosis and giardiasis in economic poultry (laying and meat chickens, ducks, and geese) may lead to extensive economic losses [61,62]. A prevalence of 13.1% of *Cryptosporidium* spp. was found from 47 quail farms in China, where the predominant species was *C. baileyi,* generally associated with the respiratory form of cryptosporidiosis in birds and capable of infecting a variety of avian hosts [63]. As regards public health concerns, the zoonotic species *C. parvum* was detected on a large turkey farm and post slaughter [64]. Several studies also investigated wild birds’ infection with *Cryptosporidium* [20,21,22,61,65]. Some of them demonstrated the presence of *C. parvum* in wild birds, suggesting a potential important role of infected birds in its spreading and transmission [21,65]. Experimental as well as field evidences of mechanical transmission of *Cryptosporidium parvum* and *C. hominis* to water by birds’ feces exist [4].

Similarly, birds can act as reservoir hosts as well as mechanical vectors of *Giardia* [4]. This parasite has an extensive zoonotic reservoir and the cysts of assemblages virulent to humans are common in water, where they can retain infectivity for two months [66], and can be acquired by birds from this environment [4]. The zoonotic *G. duodenalis* assemblages, A and B have been reported in birds [20,22,65].

Beside cryptosporidiosis and giardiasis, also microsporidiosis is a serious human disease, mainly of waterborne origin. The transmissive stages (spores) are environmentally robust and therefore ubiquitous in aquatic habitats [67]. Microsporidia can enter surface, drinking and recreational water resources from aquatic birds [4]. The most relevant zoonotic species are *Enterocytozoon bieneusi*, *Encephalitozoon intestinalis*, *Encephalitozoon hellem* and *Encephalitozoon cuniculi* [4]. In particular, *E. bieneusi* and *E. intestinalis* are the most common zoonotic species worldwide, mainly found as responsible for chronic diarrhea in HIV-infected patients, but also of acute, self-limiting diarrhea in immunocompetent persons. *Encephalitozoon cuniculi* and *Encephalitozoon hellem* have been mainly described in immunocompromised patients as agents of local (e.g., ocular) or disseminated infections [25].

The zoonotic species which was found in this study in two pigeons, *E. hellem,* is known to be able to infect birds, and it was found in *Anas platyrhynchos*, *Anser anser, Cygnus olor*, *Cygnus atratus*, *Cygnus malanocoryphus*, *Corvus corone, Melopsittacus undulates, Coscoroba coscoroba*, *Balearica pavonina* in Poland [28], as well as in *C. livia* from hurban parks in Spain [29] To the best of our knowledge, this microsporidian species had not been reported in pigeons from Italy before. The presence of human-virulent microsporidia species, particularly *E. bieneusi* but also *E. hellem*, in urban pigeons has been reported worldwide, highlighting a potential public health risk [29,30,31,68,69].

Cases of *E. hellem* infections in birds are frequently asymptomatic, but non-specific clinical symptoms may appear, often following immunosuppressive infection, inadequate husbandry, or immaturity [70,71]. The clinical picture as well as the necropsy findings in different types of birds were described in details in Snowden and Phalen [71]: depression, decreased appetite, and weight loss are most commonly reported, while stunting and increased mortality were described in nestlings. Cases of keratoconjunctivitis were also reported in companion birds [72,73]. At necropsy, significant muscle wasting, a loss of body fat and lesions mainly in the kidney, liver, intestines, and eye are found [71].

## 5. Conclusions

Although the prevalence rates found in the present survey were quite low, wild birds, with their feces, are potential source of bacterial and parasitic pathogens which can represent a threat for humans and other animals. Stantial and migratory birds may harbor some of these microorganisms in their intestinal tract without developing a disease, so they can contaminate different environments and become source of infection for mammals and other birds. On the other hand, wild birds contract bacteria and parasites from the environment, thus the spreading of pathogens among wild avifauna is also related to the diffusion of the microorganisms in other animal populations.

## Figures and Tables

**Table 2:** Positive results for at least one pathogen.

Scheme ID	Bird Species	Detected Pathogen	Method
I_52	*Larus michahellis*	*S. enterica* serovar Coeln	Isolation and typing
I_77	*Anas penelope*	*Coxiella burnetii*	PCR
I_107	*Columba livia*	*Encephalitozoon hellem*	PCR and sequencing
I_117	*Columba livia*	*Encephalitozoon hellem*	PCR and sequencing

## Data Availability

The data presented in this study is contained within the article.
